# Hypertrophic Cardiomyopathy Through the Lens of Mitochondria

**DOI:** 10.3390/biomedicines13030591

**Published:** 2025-02-28

**Authors:** Tatiana V. Kirichenko, Ivan V. Zhivodernikov, Maria A. Kozlova, Alexander M. Markin, Vasily V. Sinyov, Yuliya V. Markina

**Affiliations:** 1 Petrovsky National Research Centre of Surgery, 119435 Moscow, Russia; 2 Petrovsky Medical University, 119435 Moscow, Russia; 3 Chazov National Medical Research Center of Cardiology, 121552 Moscow, Russia; 4 Medical Institute, Peoples’ Friendship University of Russia Named After Patrice Lumumba (RUDN University), 117198 Moscow, Russia

**Keywords:** hypertrophic cardiomyopathy, mitochondrial dysfunction, oxidative stress

## Abstract

The mechanisms of pathogenesis of hypertrophic cardiomyopathy are associated with mutations in the sarcomere genes of cardiomyocytes and metabolic disorders of the cell, including mitochondrial dysfunction. Mitochondria are characterized by the presence of their own DNA and enzyme complexes involved in oxidative reactions, which cause damage to mitochondrial protein structures and membranes by reactive oxygen species. Mitochondrial dysfunctions can also be associated with mutations in the genes encoding mitochondrial proteins and lead to a violation of protective functions such as mitophagy, mitochondrial fusion, and fission. Mutations in myofibril proteins can negatively affect mitochondria through increased oxidative stress due to an increased need for ATP. Mitochondrial dysfunction is associated with impaired ATP synthesis and cardiac contractility, leading to clinical manifestations of hypertrophic cardiomyopathy. The current review was designed to characterize the role of mitochondria in the pathogenesis of hypertrophic cardiomyopathy based on published data; the search for publications was based on the analysis of articles including the keywords “hypertrophic cardiomyopathy, mitochondria, dysfunction” in the PubMed and Scopus databases up to January 2025.

## 1. Introduction

Cardiomyopathies are a heterogeneous group of genetically determined diseases affecting the heart tissue and associated with cardiac dysfunction due to myocardial hypertrophy or dilation. The following types of cardiomyopathies are distinguished: dilated, hypertrophic, restrictive, and arrhythmogenic right ventricular cardiomyopathy. Cardiomyopathies are characterized by impaired contractile function of the heart due to the molecular pathology of cardiomyocytes [1]. Hypertrophic cardiomyopathy (HCM) is the most common primary cardiomyopathy caused by genetic factors, with an incidence of 1:500–1:200 in all ethnic groups [2]. In 1990, the first gene, mutations that cause the disease, was discovered—the gene of the sarcomere beta-myosin heavy chain (MYH7). Since then, it has been believed that HCM is primarily a disease of the sarcomere, but as it turned out later, the genetic basis can be diverse. To date, about a hundred known disease genes have been identified that are expressed in various subcellular systems responsible for transcription, heart development, energy utilization, and others [3]. In most cases, HCM is clinically expressed in left ventricular (LV) myocardial hypertrophy (LV wall thickness ≥15 or ≥13 mm in familial cases) [4]. Along with LV myocardial hypertrophy, right ventricular hypertrophy may be observed, often asymmetrical, due to the thickening of the interventricular septum [5]. Myocardial thickening in HCM reduces the ability of the LV to relax and fill with blood during diastole. This leads to impaired diastolic filling of LV, decreased stroke volume and reduced ejection fraction [6]. In some cases of HCM, a thickened interventricular septum may obstruct the outflow of blood from the LV, leading to LV outflow tract obstruction and obstructive HCM [7]. The pathophysiology of HCM is multifactorial, involving a complex interaction of genetic, cellular, and structural changes leading to myocardial hypertrophy and associated metabolic abnormalities, causing clinical manifestations such as heart failure, cardiac arrhythmias, and sudden death [8].

The current review summarizes knowledge about the key mechanisms of mitochondrial dysfunction contribution to HCM that, along with impaired energy production, include oxidative stress leading to damage of cellular components, including lipids, proteins, and DNA, contributing to cardiomyocyte injury; metabolic remodeling, in particular, a shift from fatty acid oxidation to increased glucose metabolism; alteration of calcium homeostasis; changes in mitochondrial biogenesis; activation of apoptotic pathways, leading to increased cardiomyocyte death. The review also describes the possible effects of genetic mutations in sarcomeric proteins associated with HCM on mitochondrial function and their indirect influence on mitochondria through altered cellular signaling pathways.

First of all, HCM is associated with mutations in the genes encoding sarcomere proteins responsible for the contraction of cardiomyocytes, namely myosin (gene *MYH7*, OMIM 160760), myosin-binding protein C (gene *MYBPC3*, OMIM 600958), actin (gene *ACTC1*, OMIM 102540), troponin (genes *TNNI3*, OMIM 191044; *TNNT2*, OMIM 191045; *TNNC1*, OMIM 191040), desmin (*DES*, OMIM 125660). These mutations lead to dysfunction of the myocardial contractile apparatus, changes in the cardiac ejection fraction, and compensatory myocardial hypertrophy [9]. At the cellular level, these mutations lead to disruption of signal transmission in cardiomyocytes, changes in intracellular calcium content, and disruption of energy metabolism. Mitochondria perform an important function in the pathogenesis of HCM through their participation in the production of energy, reactive oxygen species (ROS) and calcium deposition [10]. Cardiomyocytes have high energy needs, and disruptions in ATP production affect the function of cardiac contraction. Mitochondrial dysfunction is also reflected in the excessive formation of ROS, mostly generated in the process of oxidative phosphorylation. High concentrations of ROS can lead to oxidative stress and mitochondrial DNA (mtDNA) damage and contribute to the development of myocardial hypertrophy, fibrosis and cell death [11]. The ability of mitochondria to store calcium is necessary for normal myocardial contraction, and disruption of calcium homeostasis is one of the causes of mitochondrial dysfunction [12]. Dysfunctional mitochondria can secrete proapoptotic factors such as Bcl-2 proteins (Bax, Bak), APAF-1 protein, cytochrome c, procaspases, and AIF protein, triggering apoptotic cascades and contributing to the loss of cardiomyocytes in HCM. Excessive cell death may further exacerbate myocardial remodeling and dysfunction of the hypertrophied heart.

The study of the complex mechanisms of mitochondria’ contribution to the development of HCM is not only of fundamental importance but also necessary for developing new therapeutic approaches to HCM. Despite the fact that the association between genetic mutations and mitochondrial dysfunction is not currently well understood, targeting mitochondrial dysfunction is a potential perspective for the treatment of HCM since there are no effective treatment strategies nowadays. The current review was designed to characterize the role of mitochondria in the pathogenesis of hypertrophic cardiomyopathy based on published data; the search for publications was based on the analysis of articles including the keywords “hypertrophic cardiomyopathy, mitochondria, dysfunction” in the PubMed and Scopus databases up to January 2025.

## 2. The Influence of Contractile Apparatus Gene Mutations on Mitochondrial Function in HCM

Myofibrils providing muscle contraction are characterized by regularly repeating structures of several groups of proteins located in contractile units, or sarcomeres. Studies of sarcomere assembly have led to the understanding that myofibrils containing mutated proteins of sarcomeres function aberrantly, which is observed in most cases of HCM [13,14,15,16]. These gene mutations can lead to disruption of the structure and function of sarcomere proteins, which leads to myocardial thickening observed in HCM. Mutations are most often found in proteins that are the main participants of the actomyosin system. Up to 75% of HCM cases caused by mutations in sarcomeric protein genes are due to *MYH7* and *MYBPC3*, 40 and 35%, respectively [17,18,19]. The β-myosin heavy chain and myosin-binding protein encoded by the *MYH7* and *MYBPC3* genes create sliding of actin and myosin filaments due to the flexibility of the myosin head and provide muscle contraction with the participation of Ca-dependent ATPase. A smaller number of cases of HCM (1–10%) are due to mutations in genes encoding other sarcomeric proteins such as actin, troponins, etc. The most common among them are mutations in *TNNT2*, which encodes the cardiac protein troponin T binding calcium ions; *TNNI3*, the troponin I gene, and *ACTC1*, the α-actin protein and may be associated with both hypertrophic and dilated cardiomyopathy [20,21]. In addition, it has been shown that a combination of sarcomeric variants in genes that determine cardiomyopathy with mutations in other genes associated with cardiovascular pathology leads to the progression of HCM and heart failure [22]. The presence of a mutant protein does not always lead to disruption of the function of microfilaments in cardiomyocytes. The study of the cardiac muscle of patients and laboratory animals indicates the dependence of pathological manifestations on the protein quality control system in the heterozygous state and mosaicism in different cardiomyocytes. The level of expression of the heterozygous mutant gene was associated with different phenotypes in animals with HCM, in which higher levels of mutant protein coincided with a more severe form of the disease [23,24]. Rare cases of severe forms of HCM are observed in patients with homozygous mutations. In addition to the secretion of mutant proteins, increased sensitivity of microfilaments to Ca^2+^ and decreased activation have been described in HCM [25]. Defective sarcomere proteins in cardiomyocytes lead to increased ATP consumption and mitochondrial load. A single point mutation *R403Q* in the human β-myosin heavy chain of cardiomyocytes causes a severe form of familial HCM [26]. Studies of human tissues with the *R403Q* mutations showed faster cross-bridge separation, increased relaxation kinetics of cardiomyocyte myofibrils, and high ATP costs for generating muscle contraction of the same force as in controls without the mutation [27,28].

Mutations in genes encoding myocardial contractile proteins are the cause of 2/3 of HCM cases, leading to structural and functional disorders of cardiomyocytes, decreased contraction efficiency and, accordingly, myocardial hypertrophy. The cell cytoskeleton is closely interconnected with the localization of mitochondria. Mutations in sarcomere genes can also affect mitochondrial function and contribute to mitochondrial dysfunction in HCM. It has been shown that cytoskeleton disorganization in permeabilized cardiomyocytes disrupts the arrangement of mitochondria, which is important for continuous ATP production. In addition, other cytoskeleton elements are also involved in the attachment of mitochondria to sarcomeres, such as desmin, the muscle-specific intermediate filament protein that was previously studied in cardiomyopathy research [29,30]. Mutations in *DES*, encoding desmin, have been identified in different kinds of cardiac and skeletal myopathies [31]. Since sarcomere function depends not only on a constant supply of ATP but also calcium, and mitochondria are calcium reservoirs that maintain the required calcium concentration, some researchers represent the sarcoplasmic reticulum, sarcomere, and mitochondria as a single contractile functional unit [32]. Mutations in sarcomere genes can disrupt ATP production, which leads to increased oxidative stress and damage to intracellular structures [33]. Mitochondrial fragmentation or swelling in cardiomyocytes carrying mutations in sarcomere protein genes indicates a potential link between sarcomere defects and mitochondrial dysfunction. Such mutations can also alter glucose and fatty acid metabolism [34]. Studies in cellular models of HCM, in particular in induced pluripotent stem cell-derived cardiomyocytes, demonstrate metabolic remodeling and calcium handling abnormality associated with the alteration of myocardial sarcomere structures as well as an increased number of damaged mitochondria and impaired mitochondrial respiration [35].

Mutations in genes encoding sarcomere proteins can affect mitochondrial function through several mechanisms, including direct interactions between sarcomere proteins and mitochondrial changes in the cell. Mitochondrial dysfunction associated with abnormal organization of mitochondria in relation to myofibrils is believed to be a key factor in pathological myocardial remodeling in HCM [36]. Mutation-induced and, consequently, mitochondrial dysfunction-induced myocardial energy depletion triggers structural and metabolic changes that cause diastolic dysfunction and cardiac hypoperfusion and, subsequently, the clinical symptoms of HCM.

## 3. The Morphology of Mitochondria in HCM

The normal structure of mitochondria is a necessary condition for maintaining their functions and is one of the important diagnostic indicators of cell function. Mitochondria are dynamic, constantly dividing and merging with each other, covering the energy needs of the cell and eliminating the effects of DNA damage [37]. The balance between the processes of fission and fusion of mitochondria, which is called mitochondrial dynamics, regulates many cellular functions. Violation of these processes is associated with the development of various diseases. Mitochondria demonstrate significant variability in shape and size. The shape can vary from almost spherical to tubular, forming networks that can change mitochondria location to reach areas that require high energy costs. Thus, the study of structural changes in mitochondria provides an understanding of the functional state of the cell in health and disease [38].

**Figure 1:**
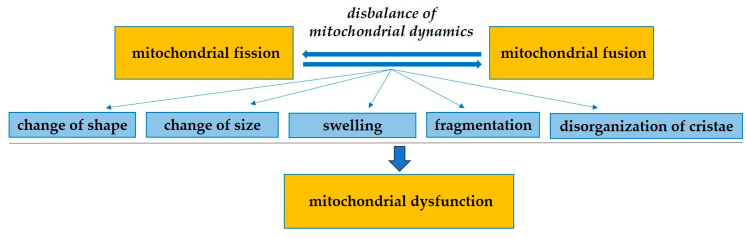
Structural changes of mitochondria in HCM.

**Figure 2:**
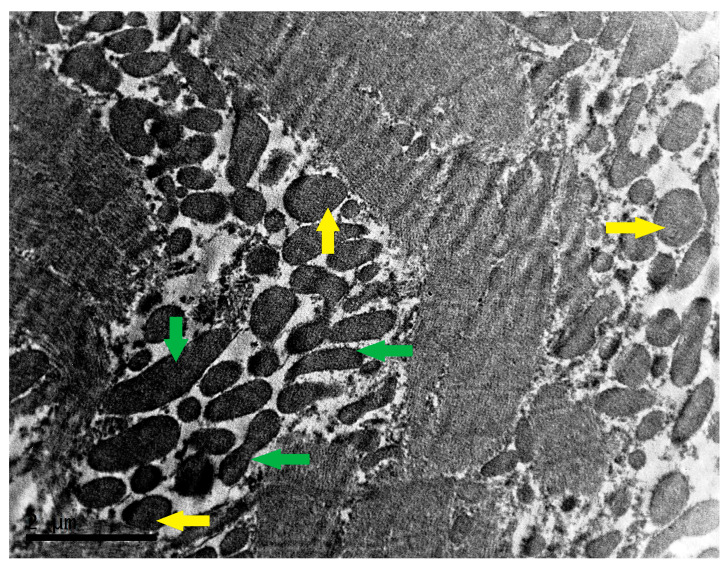
Mitochondria in the myocardium of a patient with HCM. Large interfibrillar mitochondrial cluster in a cardiomyocyte of a patient with HCM. Mitochondria demonstrate hyperchromy due to the dense packing of cristae, which vary considerably in size and shape with the presence of spherical (yellow arrows) and tubular (green arrows). Transmission electron microscopy, ×14,000. The scale bar represents 2 µm.

Figure 1 demonstrates the basic structural changes of mitochondria in HCM. Swelling and fragmentation of mitochondria in HCM indicate a violation of mitochondrial fusion. The remodeling and disorganization of the cristae, structures of the inner mitochondrial membrane that contain membrane-bound enzyme complexes involved in ATP synthesis, are also observed in cardiomyocytes in HCM [39,40]. In addition, cardiomyocytes in HCM are characterized by changes in the distribution and localization of mitochondria within the cell, which is important for the production of ATP near sarcomeres. Figure 2 demonstrates small, polymorphic, markedly hyperchromic mitochondria with increased packing density of the mitochondrial cristae in the myocardium of a patient with HCM.

The study of the diagnostic significance of mitochondrial structural changes is one of the most important research areas in HCM. Using confocal microscopy, the changes in mitochondria structure may be visualized, and the identification of structural abnormalities may indicate mitochondrial dysfunction in patients with HCM [41]. At the same time, it was shown in 59 patients with genetic hypertrophic cardiomyopathy that mitochondrial dysfunction assessed by respirometry with investigation of oxidative phosphorylation and fatty acid oxidation in septal myectomy tissue was not associated with reduced abundance or fragmentation of mitochondria evaluated by transmission electron microscopy [36]. Defects in mitochondrial morphology in HCM may indicate impaired ATP production and calcium metabolism, ROS generation, and apoptosis signaling. These disturbances may affect mitochondrial function and contribute to the pathogenesis of HCM by alteration of energy metabolism and activation of oxidative stress [42]. Evaluation of mitochondrial structural changes in myocardial samples from patients with HCM may provide diagnostic information about the degree of mitochondrial dysfunction and its role in disease progression. Quantitative analysis of mitochondrial parameters such as ratio, shape, and number may help to characterize mitochondrial changes and associated clinical outcomes in HCM [43]. Targeting mitochondrial membrane proteins and lipids may represent new strategies to manage mitochondrial dysfunction in HCM [44].

## 4. The Disfunction of Mitochondria in HCM

One of the main functions of mitochondria is the production of energy in the form of ATP. Dysfunction of mitochondria can lead to impaired ATP synthesis and increased oxidative stress. It was shown in several studies that patients with HCM often have mitochondrial abnormalities, including decreased ATP production and increased ROS concentrations [41]. These changes can contribute to the development and progression of HCM. It has been established that some mutations in mtDNA genes can contribute to the development of HCM [45]. For example, a case report describes a case of hypertrophic cardiomyopathy due to mtDNA mutation m.3303C > T in the *MT-TL1* (OMIM 590050) gene in a 35-year-old male [46]. Mutations m.3260A > G in the *MT-TL1* gene, several mutations in the mitochondrial tRNA genes (*MT-TG* (OMIM 590035), *MT-TK* (OMIM 590060), *MT-TI* (OMIM 590045)), as well as in the genes encoding mitochondrial proteins (*MT-CYB* (OMIM 516020), *MT-ATP8* (OMIM 516070)) were identified, which lead to defects in the ultrastructure and functions of mitochondria [47].

**Figure 3:**
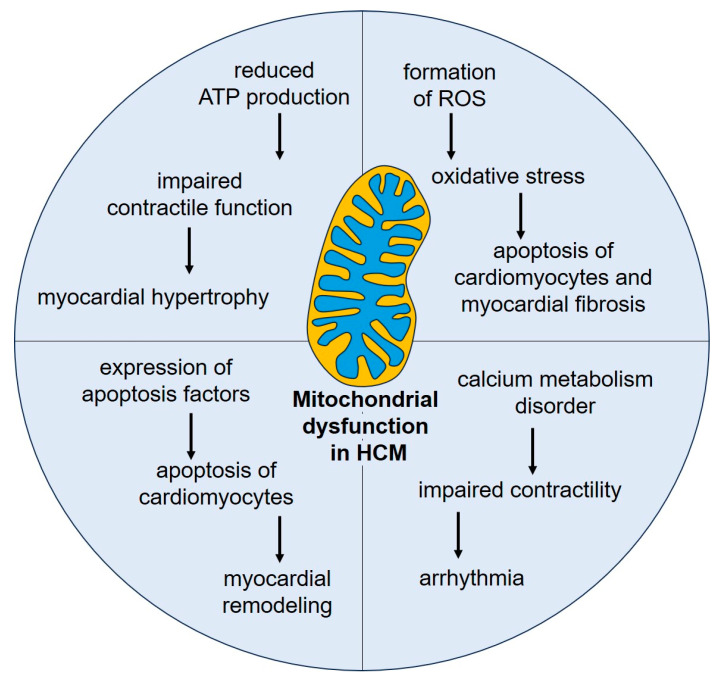
The role of mitochondrial dysfunction in the pathogenesis of HCM. ATP—adenosine triphosphate; ROS—reactive oxygen species.

Changes in the functioning of enzyme complexes and energy metabolism are important factors in HCM progression [48,49]. The electron transport chain (ETC) is a system of protein complexes located on the inner mitochondrial membrane that provides the process of electron transfer for ATP synthesis with the participation of the energy of the proton gradient. Dysfunction of the ETC, especially complex I and complex IV, contributes to impaired mitochondrial respiration and is observed in patients with HCM [50,51]. Measurement of oxygen consumption rate is a method used to assess mitochondrial respiration in cells and tissues. It was shown that HCM is associated with a decreased oxygen consumption rate, indicating impaired mitochondrial respiration in the hypertrophied heart. Changes in mitochondrial metabolism, such as a shift from fatty acid oxidation to glycolysis, have been reported in HCM [52]. Dysregulation of mitochondrial metabolism affects cardiac function and contributes to the development of myocardium hypertrophy and heart failure in patients with HCM. Figure 3 shows the main mechanisms of mitochondrial dysfunction in the pathogenesis of HCM, which are discussed below.

Although mitochondrial dysfunction has been implicated in the pathogenesis of HCM, mutations in mitochondrial protein genes have not been as well studied as mutations in sarcomere proteins. Detection of mtDNA mutations in patients with HCM is a challenge due to the phenomenon of heteroplasmy and variability in mitochondria phenotype [53,54]. Patients with HCM associated with mtDNA mutations may present with a number of clinical features, including myocardial hypertrophy, arrhythmia, heart failure, and other cardiovascular complications [55]. Disease severity and progression may vary depending on the specific mtDNA mutation and its impact on mitochondrial function. Mutations in nuclear genes encoding proteins involved in mitochondrial function, such as proteins participating in mitochondrial biogenesis, fusion-fission dynamics, or respiratory chain complexes, can also lead to mitochondrial dysfunction in HCM. For example, mutations in genes encoding proteins such as PINK1, Parkin, or OPA1 have been associated with mitochondrial dysfunction [56,57]. Mutations in nuclear genes involved in mitochondrial RNA metabolism, such as *ELAC2* (OMIM 605367) gene coding for the mitochondrial RNase, are associated with HCM, mitochondrial respiratory chain deficiency, and lactic acidosis [58]. In mouse models deleting the mitofusins *Mfn1* (OMIM 608506) and *Mfn2* (OMIM 608507), proteins responsible for mitochondrial outer membrane fusion, muscle atrophy, mitochondrial dysfunction, and compensatory increased mitochondrial proliferation were observed. These cells consume less oxygen and have low activity of respiratory complex 1 compared to healthy cells. In addition to mitofusins, a dynamin-like protein, optical atrophy protein 1 (OPA1), which is involved in the fusion of internal membranes and the organization of mitochondrial cristae, has also been characterized [59,60,61]. Studies on cell and mouse models have shown that the effect of the drug 1-deoxynojirimycin on the OPA1 protein normalizes the function of cardiomyocyte mitochondria and reduces cardiac hypertrophy, which may have great potential in the development of therapeutic drugs for the treatment of HCM. The mechanism of this effect is the oligomerization of OPA1, which leads to the restoration of mitochondrial cristae [62].

An important function of mitochondria is their participation in calcium homeostasis. Mutations in the genes of proteins involved in calcium metabolism can contribute to the development of HCM [63]. Disruption of calcium signaling in mitochondria leads to a deficiency or excess of calcium concentration in the sarcoplasm and impaired cardiomyocyte contraction [64]. It was shown in a cell model of cardiomyopathy, a culture of cardiomyocytes derived from induced pluripotent stem cells from a patient with HCM carrying a *MYH7* mutation, that inhibition of myofilament calcium sensitivity with mavacamten significantly ameliorated cardiomyocyte hypertrophy [65]. These data suggest that enhanced myofilament calcium sensitivity is an important mechanism in the pathogenesis of HCM. Increased oxidative stress is also a sign of mitochondrial dysfunction, and mutations in genes involved in antioxidant defense mechanisms can aggravate oxidative damage and contribute to the development of HCM [41]. Genes encoding antioxidant enzymes, such as superoxide dismutase, catalase or glutathione peroxidase, may be involved in this process [66]. Patients with HCM have increased activity of mitochondrial complex I and antioxidant superoxide dismutase [67]. A recent case report describes a patient with HCM due to mitochondrial assembly factor gene *NDUFAF1* (OMIM 606934) biallelic variants associated with mitochondrial complex I deficiency [68]. In another study, a case of severe HCM in a neonate carrying a mutation in the mitochondrial complex I gene, *NDUFB11* (OMIM 300403), was demonstrated [69]. The contribution of genetic variants associated with HCM in the development of mitochondrial dysfunction is summarized in Table 1.

Dysfunctional or damaged mitochondria are normally selectively removed to maintain cellular homeostasis. This process is called mitophagy, which is considered one of the main mechanisms to control the quality of mitochondrial functioning [70]. There are ubiquitin-dependent and ubiquitin-independent mitophagy pathways. Among the ubiquitin-dependent pathways, the most widely studied is the PTEN-induced kinase 1 (PINK1)/Parkin pathway, which is involved in the elimination of damaged mitochondria in mammals. PINK1 is a highly conserved mitochondrial protein encoded by the *PARK6* (OMIM 605909) gene and is involved in the regulation of mitochondrial function. In normal mitochondria, PINK1 expression on the outer mitochondrial membrane is low since it is constantly transferred to the inner membrane and cleaved [71]. When the membrane potential deviates from the normal range, the mechanism of PINK1 entry into the inner mitochondrial membrane is disrupted, resulting in PINK1 accumulation in the outer mitochondrial membrane. Once PINK1 accumulates and stabilizes on the outer mitochondrial membrane, it activates the E3-Ub ligase Parkin through a mechanism involving phosphorylation of Parkin and its substrate Ub at Ser65 (pSer65-Ub). Sufficient accumulation of pSer65-Ub in the outer mitochondrial membrane can trigger the recruitment of the autophagy receptors optineurin (OPTN) and nuclear dot protein 52 (NDP52), which can promote the initiation of autophagy near the mitochondria. Less studied ubiquitin-independent pathways are associated with proteins (NIX)/BCL2, FUNDC1, and MARCH5, which also function via outer membrane expression [72]. Dysregulation of mitophagy in HCM may lead to the accumulation of defective mitochondria, which is accompanied by oxidative stress and accumulation of oxygen radicals, exacerbating mitochondrial dysfunction [73].

Mitochondrial dysfunction in HCM may result from a complex interaction of genetic and environmental factors affecting mitochondrial structure and function. Although mutations in nuclear genes encoding sarcomere proteins are the predominant genetic cause of HCM, mtDNA mutations may also play a role in the pathogenesis of the disease. Among mitochondrial disorders that lead to cardiac dysfunction, the most studied are disorders of mitochondrial fission and fusion, as well as mitophagy. Understanding the impact of mtDNA mutations on mitochondrial function and cardiac physiology is important for comprehensive genetic assessment and individual management of individuals with HCM. Therapeutic strategies aimed at correcting mitochondrial dysfunction, in particular the use of antioxidants to reduce oxidative stress or improving mitochondrial function through pharmacological interventions, may be a promising approach in the pathogenetic therapy of HCM [44,74].

## 5. Mitochondria-Targeted Therapeutic Strategies in HCM

Mavacamten (MYK-461) is a novel pharmacological agent targeting the sarcomere by modulating the β-MHC, which is the most widely-used and well-studied preparation designed for the symptomatic treatment of cardiomyopathy [75]. It can be assumed that improving heart function with Mavacamten can potentially affect the mitochondria since mitochondria play a key role in the energy metabolism of cardiomyocytes. However, the effects of Mavacamten on mitochondrial dysfunction haven’t been studied yet.

Normalization of mitochondrial function is a potential therapeutic strategy for the treatment of HCM. The use of antioxidants and modulators of mitochondrial dynamics preparations aimed at energy metabolism correction have shown promising results in preclinical and clinical studies and, therefore, may contribute to the treatment of HCM by correcting mitochondrial dysfunction. Several strategies aimed at maintaining mitochondrial function in HCM, presented in Table 2 are currently being explored.

### 5.1. Mitochondrial Biogenesis Stimulators

The activation of the formation of new mitochondria can help improve mitochondrial function and ATP production in cardiomyocytes. In particular, the coactivator of peroxisome proliferator-activated receptor gamma 1-alpha (PGC-1α) stimulates mitochondrial biogenesis by regulating genes involved in fatty acid oxidation and oxidative phosphorylation. One such activator is bezafibrate, a lipid-lowering drug that has shown efficacy in muscle atrophy and encephalopathy in mice, as well as in rat models of neurodegenerative diseases due to mitochondrial protection [76,77]. Clinical studies of bezafibrate have also been conducted to assess its efficiency in mitochondrial trifunctional protein deficiency (TFP). It was demonstrated that bezafibrate administration led to improvement in fatty acid oxidation disorders in TFP-deficient patient fibroblasts and reduction of myopathic manifestations in patients with TFP deficiency [78,79]. Among PGC-1α activators, urolithin and empagliflozin also have been shown to improve mitochondrial function [80].

### 5.2. Modulators of Mitochondrial Dynamics

Restoring the balance between mitochondrial fusion and fission is critical for maintaining mitochondrial function. Modulators of mitochondrial dynamics, such as mitochondrial fission inhibitor-1 (Mdivi-1) or P110, may contribute to the normalization of mitochondrial structure and function in HCM. P110 inhibits the interaction of dynamin-like protein-1 (DRP1) with its adaptor Fis1, which is required for mitochondrial fission, while Mdivi-1 can suppress mitochondria-associated apoptotic pathways [81,82]. Regular exercise has been shown to stimulate mitochondrial fission and improve cardiac health in HCM [83]. In aged mice, exercise significantly increased levels of the protein DRP1, which is essential for mitochondrial fission, had a positive effect on mitochondrial cristae structure, and increased ATP production [84].

### 5.3. Mitophagy Modulators

Enhancement of mitophagy, the selective removal of damaged mitochondria, can help eliminate dysfunctional mitochondria and decrease ROS levels in the cell. Several studies demonstrate that mitophagy inducers such as rapamycin or urolithin A promote the removal of defective mitochondria and reduce oxidative stress in cardiomyocytes [85]. Astaxanthin stimulates mitophagy by increasing the expression of *PINK1*, *Parkin*, and mtDNA genes, thereby reducing vascular remodeling caused by hypertension [86]. The most promising modulators of mitophagy are inducers of the *PINK1/Parkin* signaling pathway, which are the most studied to date. Such inducers include kinetin triphosphate (KTP) and its precursor kinetin. KTP has a higher affinity for PINK1 than ATP, thereby activating PINK1 and increasing its affinity for Parkin, which is necessary to initiate mitophagy [87]. Suppression of mitophagy inhibitors is also considered an effective treatment strategy. For example, pifithrin-α suppresses p53, a transcription factor regulating the cell cycle that binds to Parkin and blocks its interaction with PINK1, which explains the mechanism of the inverse dependence of apoptosis on mitophagy [88]. Pharmacological regulation of mitophagy requires a delicate approach depending on the stage of disease development since cardiomyocytes have a low capacity for proliferation.

### 5.4. Antioxidants and Metabolic Modulators

Mitochondrial dysfunction in HCM is often associated with increased oxidative stress, leading to damage to mitochondrial components and impaired function. Antioxidants such as MitoQ or elamipretide (SS-31) may help reduce oxidative stress in mitochondria, protect against damage, and improve mitochondrial function in HCM [36]. Targeting metabolic pathways such as glucose utilization or fatty acid oxidation with preparations such as trimetazidine or perhexiline can optimize energy substrate utilization and improve mitochondrial function in the hypertrophied heart [89]. When HCM is caused by impaired transport or oxidation of long-chain fatty acids, triglycerides may be an effective alternative energy substrate. Triheptanoin has been shown to be effective in clinical trials in patients with hypoglycemia, cardiomyopathy, and rhabdomyolysis [90]. Ketone bodies are less widely used as an alternative energy substrate in fatty acid oxidation disorders, and an analysis of several studies showed that empagliflozin reduced ventricular mass, cardiomyocyte cross-sectional area, and myocardial fibrosis in animal models by improving ketone metabolism [91]. Spermidine has been shown as a promising polyamine in a number of studies due to its modulation of mitochondrial function and fatty acid oxidation in mice [90]. Experiments on mice demonstrated cardioprotective properties of spermidine. In particular, it helps to reduce age-related changes in cardiac morphology, stimulates mitochondrial biogenesis and mitophagy, and has a positive effect on the localization of mitochondria in relation to sarcomeric myofibrils, which may be a consequence of the general improvement in mitochondrial protein metabolism [92].

Therapeutic strategies for mitochondrial dysfunction in HCM aim to restore normal mitochondrial function, reduce oxidative stress, increase ATP production, and improve overall cardiac performance. Novel gene therapy approaches targeting mitochondrial genes as well as sarcomeric genes involved in mitochondrial dysfunction in HCM are being explored as potential therapeutic strategies to restore mitochondrial health and improve cardiac function [93,94]. Further studies are needed to confirm the efficacy and safety of these approaches in clinical settings and to develop personalized therapies for patients with HCM. The efficacy and safety of many therapeutic agents have been demonstrated in other diseases with underlying mitochondrial dysfunction, such as Parkinson’s and Alzheimer’s diseases, which need to be tested in HCM models. These treatment approaches affect such processes as mitochondrial fission/fusion, mitophagy, and mitochondrial survival, and the therapeutic effect largely depends on the balance of these processes.

## 6. Conclusions

The mechanism of HCM was initially considered as associated with a defect in the genes of sarcomeric proteins in cardiomyocytes. In recent years, genetic diagnostics of heart disease using next-generation sequencing has identified genetic variants associated with cardiomyopathy in about 170 genes. The complexity of the cellular and molecular pathogenetic mechanisms makes it difficult to understand the clinical significance of most genetic sequence variants [95]. At the same time, it is known that mitochondrial dysfunction makes a significant contribution to the pathogenesis of cardiomyopathy. Unlike mutations in genes encoding sarcomere proteins, mitochondrial dysfunction has a wider range of pathogenetic effects at the molecular and cellular level, which determines a variety of potential therapeutic approaches. Understanding the relationship between mutations in sarcomere protein genes and mitochondrial dysfunction in HCM is important for the development of targeted therapeutic strategies aimed at improving cardiac function and slowing disease progression in patients with HCM. Correction of mitochondrial dysfunction and restoration of energy balance can help alleviate the metabolic disorders underlying HCM. Further studies are needed to elucidate the mechanisms underlying mitochondrial dysfunction in HCM and to explore new mitochondrial-targeted interventions. Genome-wide association studies will reveal the relationship between the genotype and the phenotype of cardiomyopathy patients, contributing to the development of cardiomyopathy treatment. The studies of existing agents and their analogs that have been shown to be effective in myopathies and neurodegenerative diseases may be a promising direction in the research aimed at the development of novel therapeutic strategies in cardiomyopathy. Studying mitochondrial respiration, metabolism, and mitochondrial mutations in the context of HCM may shed light on the role of mitochondrial dysfunction in the pathogenesis and progression of this disease and may help in the development of personalized therapeutic approaches.

## Figures and Tables

**Table 1:** The contribution of mtDNA HCM-associated genetic variants in mitochondrial dysfunction of cardiomyocytes.

Gene	Encoded Product	Mechanisms of Mitochondrial Dysfunction
*MT-TL1* (OMIM 590050)	The mitochondrial tRNA for leucine (UUR) encoded by nucleotides 3230–3304	Mitochondrial tRNA dysfunction [46]
*MT-TG* (OMIM 590035)	The mitochondrial tRNA for glycine encoded by nucleotides 9991–10058	Mitochondrial tRNA dysfunction [47]
*MT-TK* (OMIM 590060)	The mitochondrial tRNA for lysine encoded by nucleotides 8295–8364	Mitochondrial tRNA dysfunction [47]
*MT-TI* (OMIM 590045)	The mitochondrial tRNA for isoleucine encoded by nucleotides 4263–4331	Mitochondrial tRNA dysfunction [47]
*MT-CYB* (OMIM 516020)	Cytochrome b (MTCYB) is the only mtDNA-encoded subunit of respiratory Complex III	Defects in the ultrastructure and functions of mitochondria [47]
*MT-ATP8* (OMIM 516070)	Subunit 8 of mitochondrial ATP synthase (complex V) is encoded by nucleotides 8366–8572 of the mitochondrial genome	Defects in the ultrastructure and functions of mitochondria [47]
*ELAC2* (OMIM 605367)	The long form of RNase Z—endonuclease involved in tRNA biogenesis	The mitochondrial RNase metabolism disturbance [58]
*Mfn1* (OMIM 608506)	Mitofusin—MFN1	Disbalance between fusion and fission [59,60,61]
*Mfn2* (OMIM 608507)	Mitofusin—MFN2	Disbalance between fusion and fission [59,60,61]
*NDUFAF1* (OMIM 606934)	CIA30 protein in complex 1 of human mitochondrial NADH: ubiquinone oxidoreductase	Mitochondrial complex I deficiency [68]
*NDUFB11* (OMIM 300403)	p17.3 of mitochondrial complex I	Mitochondrial complex I deficiency [69]

**Table 2:** Potential therapeutic strategies aimed at correcting mitochondrial dysfunction for HCM.

Treatment Strategy	Therapeutic Agents	Therapeutic Effects
Mitophagy modulators	Rapamycin, urolithin A, astaxanthin Pifitrin-α	Activation of the PINK1/Parkin signaling pathway stimulates mitophagy Suppression of the mitophagy inhibitor p53
Modulators of mitochondrial dynamics	Mdivi-1, P110	Inhibitors of DRP1-dependent mitochondrial fission recover the balance between mitochondrial fusion and fission
Mitochondrial biogenesis stimulators	Bezafibrate, empagliflozin	PGC-1α activation enhances fatty acid oxidation
Antioxidants and metabolic modulators	Elamipretide, trimetazidine, spermidine	Activation of oxidative phosphorylation leads to reduction of oxidative stress

DRP1, dynamin-related protein-1; Mdivi-1, mitochondrial fission inhibitor-1; PGC-1α, peroxisome proliferator-activated receptor gamma coactivator 1-alpha.

## Data Availability

Not applicable.
